# Segmentation of THz holograms for homogenous illumination

**DOI:** 10.1038/s41598-024-63517-7

**Published:** 2024-06-03

**Authors:** Mateusz Surma, Mateusz Kaluza, Paweł Komorowski, Agnieszka Siemion

**Affiliations:** 1 Faculty of Physics, Warsaw University of Technology, Koszykowa 75, 00-662 Warsaw, Poland; 2 Institute of Optoelectronics, Military University of Technology, gen. S. Kaliskiego 2, 00-908 Warsaw, Poland

**Keywords:** Terahertz optics, Polymers, Imaging and sensing, Sub-wavelength optics

## Abstract

This paper investigates the feasibility of applying the hologram segmentation method for homogeneous illumination. Research focuses on improving the uniformity of the illumination obtained from diffractive optical elements in the THz range. The structures are designed with a modified Ping-Pong algorithm and a neural network-based solution. This method allows for the improvement of uniform illumination distribution with the desired shape. Additionally, the phase modulations of the structures are divided into segments, each responsible for imaging at different distances. Various segment combination methods are investigated, differing in shapes, image plane distances, and illumination types. The obtained image intensity maps allow for the identification of the performance of each combination method. Each of the presented structures shows significant improvements in the uniformity of imaged targets compared to the reference Ping-Pong structure. The presented structures were designed for a narrow band case—260 GHz frequency, which corresponds to 1.15 mm wavelength. The application of diffractive structures for homogenization of illumination shows promise. The created structures perform designed beamforming task with variability of intensity improved up to 23% (standard deviation) or 45% (interquartile range) compared with reference structure.

## Introduction

Homogeneous illumination is one of the wide range of beam shaping challenges. It generally finds application in laboratory tasks requiring full sample area analysis^1^, industrial irradiation^2^, or imaging^3^. In the terahertz (THz) range, it can be applied for specific tasks of sample illumination, e.g., for skin cancer detection^4^ or nondestructive testing^5^. In such tasks, the quality of the sample plane illumination affects the quality of the image reconstruction in the image plane. Point- or line-like illumination is commonly used to form the image after raster scanning the sample^6^, as such solutions are readily available. Illumination in the THz range is much more troublesome compared to the visible or infrared spectral ranges because of the significant coherence of many THz sources. This, in turn, results in unwanted interferences, which often mitigate the efficiency of various beam-forming tasks. This work focuses on holographic methods of reduction of such effects in the case of quasi-homogenous illumination of a 40 mm $\times$ 40 mm square.

Uniform illumination can be obtained with either passive or active approaches. The latter usually focuses on scrambling phase distribution or scanning the sample areas with, e.g., a swiveling mirror^3,7^, which implies the addition of moving elements into a setup. Depending on an application, mechanical vibrations from moving elements and total scrambling of the phase of the wavefront might be undesirable. The range of passive approaches is much more extensive. Uniform illumination can be obtained by applying optimized refractive/reflective optical setups and elements^1,8,9^ (including freeform optics), lenslet arrays^2^ or diffractive optical structures^10^. This work focuses on a diffractive approach.

Diffractive Optical Elements (DOEs) are thin optical elements allowing for the precise control of the wavefront. They introduce wavefront changes through spatial modulation of phase. To achieve that, DOEs contain a set of binary or multi-level reliefs placed on a flat substrate. The height of reliefs depends on the wavelength (frequency) of used radiation. The low height of such a structure is particularly advantageous for the THz regime where radiation sources have less power compared, for example, with optical range. It should be taken into account that the diffractive approach introduces significant chromatic aberrations, as it is related to specific design wavelength. Some methods, such as designing the diffractive element in the form of the high order kinoform^11^, provide improvement in this area. Additionally, high-order kinoforms allow one to obtain relatively thin solutions and, therefore, introduce fewer losses due to absorption in the material. Diffractive optical elements (DOEs) also provide a unique possibility of multiplexing^12–14^—especially dividing their surface into segments, each realizing different functions, such as drawing separate parts of a letter at the output plane^15^. Applying DOEs for uniform illumination was previously reported^16^. Moreover, applying DOEs for top-hat illumination showed an overall improvement in complex optical system performance^17^.

In beamforming with diffractive structures, two general subsets of methods for structure design emerge^18^: analytical transformations and numerical methods. Analytical approaches focus on finding a coordinate transformation of the input intensity distribution to the output intensity distribution. After defining the transformation, it can be realized as a phase modulation^18^. The term numerical methods encapsulates here a broad range of iterative optimization algorithms that modify the starting phase modulation in steps, working towards the improved reconstruction of the output intensity distribution.

Numerical methods allow for the design of DOEs without knowledge of the analytically calculated phase modulation forming a particular shape at a defined distance from the structure. Their advantage is the capability of obtaining the target phase modulation without prior knowledge of the analytic description of the input wavefront. Throughout the history of diffractive optical design, a large number of iterative methods have been presented. After the introduction of the Gerchberg-Saxton algorithm for phase retrieval^19^, many related methods followed. At their root, numerical methods are optimization algorithms that also lead to other approaches such as simulated annealing^20^ or genetic algorithms^21^.

This paper investigates the feasibility of applying hologram segmentation to realize homogeneous illumination in the THz range of radiation. Segmentation is applied to reduce the effects of significant coherence of a THz source. The idea of the proposed approach revolves around the mitigation of unwanted speckle noise within the uniform distribution pattern. This is a known problem in coherent optical systems, and the proposed methods of reduction of this effect aim to reduce the coherence of an illuminating beam by introducing the angle, polarization, or wavelength diversity^22^. As the latter two are infeasible in the case of the narrow-band, linearly-polarized THz emitters, we focus on the spatial approach to decrease the coherence of the input beam. We proposed the spatial segmentation of the designed DOEs. In this way, the segments of the structure act as separate holograms placed at different distances from the source. Thus, the speckle pattern for each segment is different, allowing for the mitigation of the resultant speckle noise.

The design process of the diffractive structures was based on the modified Ping-Pong algorithm. A single-plane DOE, designed with the same method, served as a reference, allowing for the verification of the influence of the segmentation on the obtained intensity pattern. Moreover, two additional structures were prepared with a previously investigated novel neural network-based algorithm^13,23^. Different approaches to segmentation and homogeneity improvement have been tested in numerical simulations. The best-performing structures have been chosen for manufacturing and experimental evaluation. The structures have been fabricated using the 3D printing method. The experimental results show a significant correlation with the simulations and suggest the potential feasibility of the method for achieving uniform illumination patterns.

## Materials and manufacturing

Diffractive structures realize a desired beam shaping by an introduction of a specifically designed distribution of the complex amplitude. It can be accomplished through modification of an amplitude (in the case of opaque elements) or phase of the optical field (for transparent objects). The phase modulation is achieved by manufacturing an optical element with thickness distribution related to the particular phase shifts. The design procedure and the type of phase delay map coding define them. In this research, an iterative optimization method with scalar propagation was used for phase modulation design. At the same stage, simulations of structure performance were obtained. Such designed phase delay maps were then 3D modeled and 3D printed.

### Structure design

Structures are designed with an iterative algorithm based on the modified Ping-Pong algorithm. This method operates within the area of scalar wave optics; therefore, it defines states of amplitude and phase at given optical planes. Two planes are defined: the hologram plane and the single image plane—the algorithm switches between those planes through modified Fresnel propagation^24^ (a modified convolution approach implemented in Light Sword 6.0 software). A number of planes can be extended^25^. At each plane, the algorithm enforces predefined amplitude distributions (a uniform amplitude for the hologram plane and a desired shape for the image plane), transferring information into phase distribution by propagation between planes. The phase distribution obtained at the end of the algorithm at the hologram plane forms a phase delay map of the diffractive element. The modified ping-pong algorithm has been chosen both for the design of the investigated structures (segmented) and the reference one (a single plane). This is a standard for designing computer-generated holograms (in visible as well as THz spectral ranges). It has been shown to significantly improve the obtained intensity patterns compared to the single backpropagation of the target intensity distribution^26,27^.

**Figure 1:**
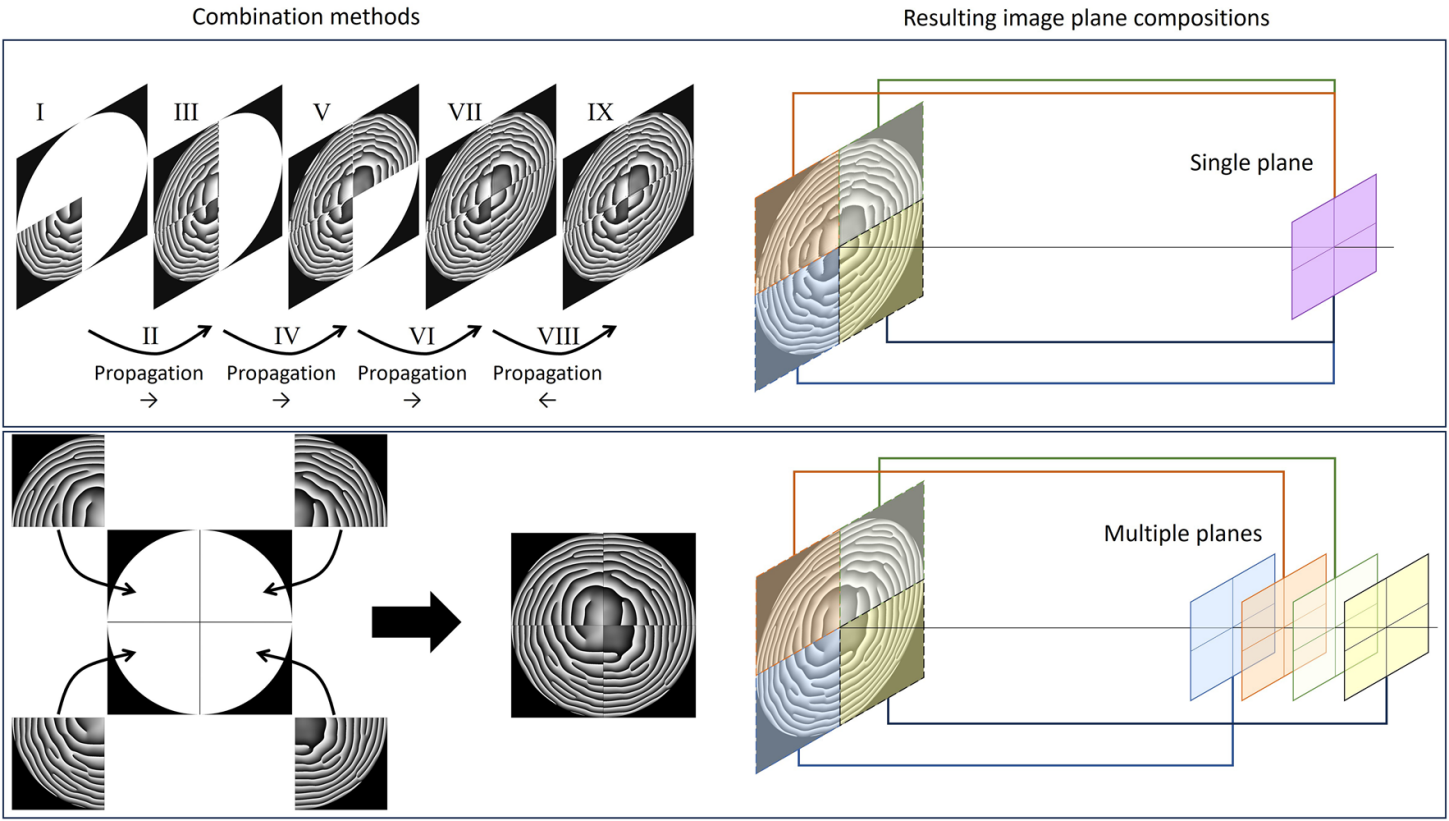
Visualization of two different combination methods resulting in single (top row) and multiple image planes (bottom row). The left side of the images shows how phase segments are combined, and the right side displays the influence of each segment on the final image plane composition. In both methods, each quarter is calculated separately, and each is responsible for imaging a square at slightly different distances (490 mm, 495 mm, 500 mm, and 505 mm). The first case introduces each quarter one by one in the order from the largest imaging distance to the smallest. Between the introduction of consecutive quarters, propagation is used to move by the difference in imaging distances to the plane of the next quarter (Roman numerals show the order of operations). The last propagation is backward to arrive at the selected distance of 500 mm from the image plane. As a result of the first method, image planes from each quarter coincide. In the second method, all quarters are placed at the same plane, resulting in separate images of a square.

**Figure 2:**
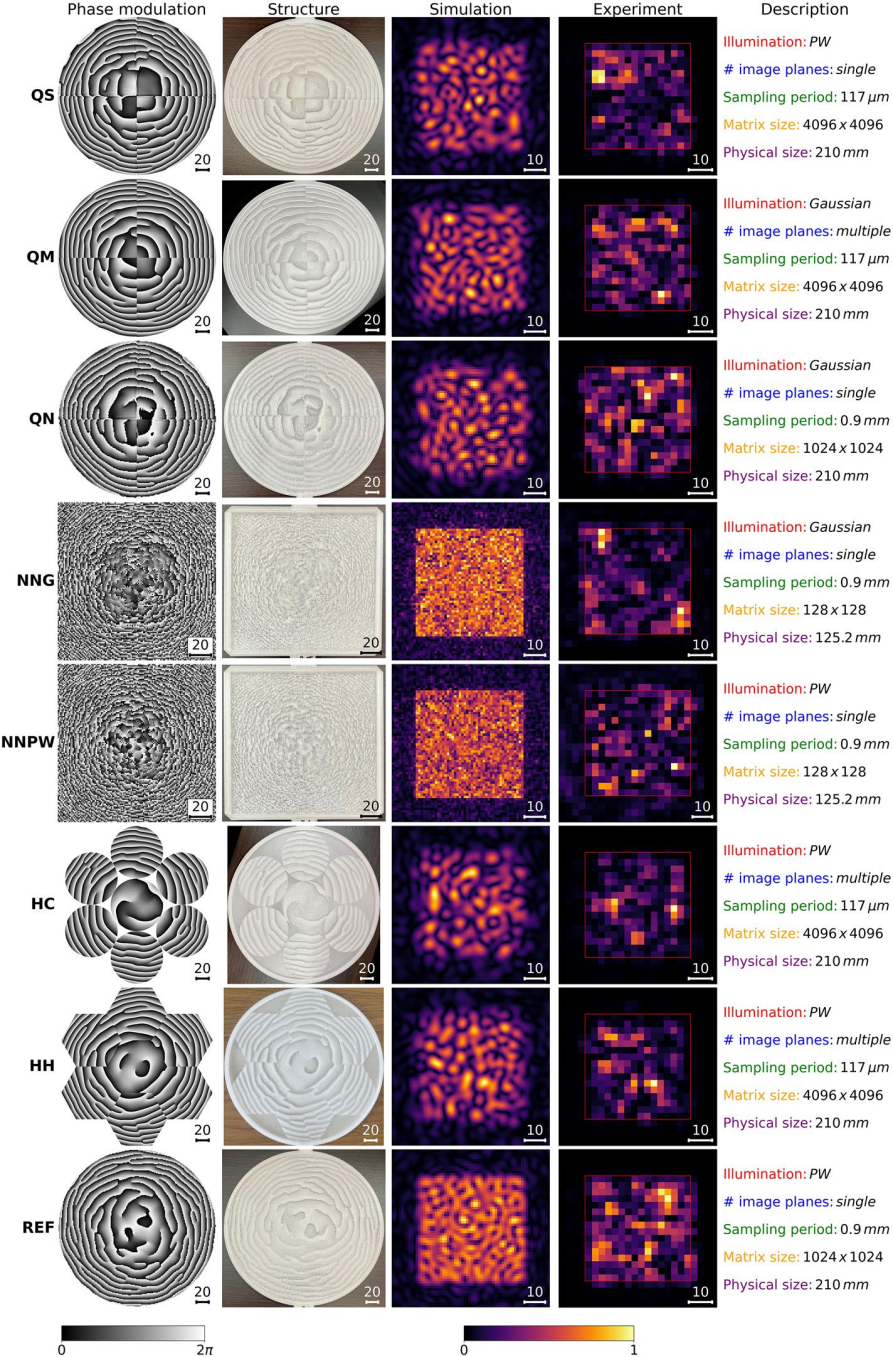
A summary of examined structures. In columns from left to right: a phase modulation introduced by each structure; in the next column, a photograph of the manufactured structure; numerical simulation results; experimental data; a short description of the properties of each structure. Acronyms on the leftmost side identify structures. Phase modulations show changes in the $0{-2}\pi$ range (black to white) with a color scale at the bottom left of the figure. Intensity distributions of simulations and experiments are separately (individually) normalized with a common color scale at the bottom right of the figure. Images from experimental evaluation contain red squares showing areas selected as a Region of Interest (ROI) for each structure. The detailed quantitative comparison of structures is given later in the “Results and discussion” section. The size scale in millimeters is placed in the bottom right corner of each image.

In this work, the phase delay map area has been divided into segments with different functionalities. For the purpose of this study, four segments were defined for each structure. All segments were imaging the same target—a square with a side of 40 mm—but at slightly different distances: 490 mm, 495 mm, 500 mm, and 505 mm. Segments may be organized in multiple ways on the structure’s surface. For example, a circular aperture may be divided into quarters, each representing a separate segment. The way the segments are combined is an additional degree of freedom in this design method. Since, in this method, each segment represents a separate part of the whole phase modulation, one can calculate each segment separately and perform scalar propagation on each of them. For this reason, phase modulation of one segment can be propagated by a small distance, and then a modulation from another can be combined. Using this concept, one can make the whole structure image at one chosen distance—single plane, even though all designed segments have different imaging distances. This method of segment combination with resulting image plane composition is depicted in the top row of Fig. 1. Another option is to simply combine all segments, imaging at different distances, into a structure. As a result, the final structure produces multiple image planes at once. The bottom row of Fig. 1 shows this combination method with the following image plane composition. The illumination type and number of image planes for each structure are also marked in Fig. 2.

The design wavelength (DWL) was fixed to 1.15 mm (corresponding to the frequency of 260 GHz), which matched the available equipment and optical properties of the materials (mainly, the absorption coefficient).

Eight types of structures are investigated in this paper: five designed with the proposed segmentation method, two designed with neural-network-based (NN-based) approach, and reference structure, all illustrated in Fig. 2. Within the set of segmented structures, three DOEs were divided into quarters (four segments of a circle) and combined to reconstruct a square at different distances (Quartered with Multiple planes—QM) or the same single distance (Quartered with Single plane—QS). The third quartered structure was combined to reconstruct at a single plane like QS but had other simulation parameters chosen to be closer to the NN-based structures (Quartered with NN-based parameters—QN). The design of this structure aimed to provide a better comparison with the NN-based structures, which will be described in detail later. The following two structures have been divided into honeycomb cells with round (Honeycomb with Circular apertures—HC) or hexagonal (Honeycomb with Hexagonal apertures—HH) apertures. The central aperture and each pair of opposing cells were treated as separate segments (resulting in a total number of four segments).

A reference structure (REF) has been designed with a standard Ping-Pong algorithm. It consists of a single segment in a single plane. It can be described as a typical computer-generated hologram for the uniform illumination of a square.

The sampling period of a model of the structure was set to 117 μm based on the requirements of the manufacturing method. Since the propagation method uses the Fourier transform, the resolution of a calculation matrix had to be large enough to avoid unwanted sampling effects. The resolution was set to $4096\times 4096$ points. The exceptions were QN and REF, whose sampling period was set to 0.9 mm and resolution was reduced to $1024\times 1024$.

Subsequently, two structures have been designed with the previously investigated algorithm for the optimization of the DOEs based on the neural network (NN)^13,23^. The structures designed with the NN algorithm do not utilize the segmentation method. They are both single-plane DOEs, consisting of a single segment and work as an additional reference, designed with a unique, novel method. The principle of the NN-based design method is described in our recent publication^23^. The crucial information is that the NN algorithm uses the convolution method to simulate radiation propagation and adaptive moment estimation (ADAM^28^) method to optimize the phase delay map to match the target amplitude (40 mm square). The optimization parameters have been set to $\alpha =0.1$, $\beta _1=0.9$, $\beta _2=0.999$ and $\epsilon =10^{-5}$. Optical design parameters were the same as for segmented structures. However, the sampling period was set to 0.9 mm and matrix resolution to $128\times 128$ points, which results from the implementation of the NN algorithm. Two structures have been designed with this method applying the illumination with a uniform amplitude (NNPW) and a Gaussian-shaped amplitude (NNG).

Two types of illumination have been investigated in the simulations. The first, denoted as plane wave (PW), is an illumination with a constant amplitude distribution. It makes sense from the perspective of the structures’ design, as every part of the element contributes equally to forming the final intensity pattern. This approach, however, is not physical, as in most cases the amplitude distribution of the illuminating field is of Gaussian shape. Therefore, some structures have been designed using the Gaussian amplitude distribution. This approach described the experiment more accurately, but it pays less attention to the optimization of the outside regions of the structure. It should emphasized that in both approaches, the illuminating wavefront is flat (the beam is collimated, forming the quasi-plane wave). The differences concern only the amplitude distribution of the beam.

Parameters and types of structures are additionally summarized in Fig. 2.

### Manufacturing

Physical models can be created from numerical representations of phase modulation distributions with the use of 3D printers. Fused Deposition Modeling (FDM), also called Fused Filament Fabrication, was chosen as the manufacturing method for all diffractive structures described in this paper. Numerical representations of the holograms have been saved in 8-bit loss-less BMP files, which means that the phase values obtained have been sampled into 256 levels. Next, 3D models were obtained from BMP files, with dimensions accounting for the known refractive index of the material used to manufacture the structure.

In the modeling process, two different approaches were performed. The first solution was the representation of each pixel by a node. Nodes were extruded into different height levels according to the phase values varying from 0 to 255 and the material’s refractive index used in manufacturing. The height represented by each pixel was calculated using the physical formula $h(x,y) = \frac{\lambda }{2 \pi } \frac{\phi (x,y)}{n-1}$, where *h*(*x*, *y*) is the extrusion pixel height as a function of *x* and *y* Cartesian coordinates, $\phi (x,y)$ is the desired pixel phase retardation distribution, $\lambda$ is the design wavelength, and *n* is the refractive index of the material. Subsequently, nodes were connected into the triangular mesh, creating a 3D model. Due to the interpolation between pixels, such an approach gives satisfying results for small sampling values and continuous phase changes of the structure. This method was performed for structures with 117 μm and $4096\times 4096$ px using Blender software.

The second approach for 3D modeling is a novel method presented in our previous study^23^. The idea is to extrude each pixel as a cuboid into the height level described by the structure’s height formula. As a result, the extruded pixels more accurately represent gray-scale BMP files. Sampling is precisely determined, allowing the 3D printer to accurately manufacture the area of each cuboid according to the nozzle size used in the manufacturing process. Thus, the gray-scale images are precisely represented by 3D models and manufactured structures. Moreover, this approach allows for a more accurate representation of irregular and chaotic phase distributions that might occur, e.g., in NN-based algorithms. This modeling method is a better solution for structures with smaller matrix sizes and sampling distances larger than 900 μm, and it was applied for structures QN, REF, NNPW, and NNG.

The designed thickness of each structure was 2.07 mm, as calculated by the formula for the height of the structure. Since phase modulation could locally have a zero value, we included a 1.5 mm substrate for all structures, resulting in a total maximum thickness of 3.57 mm. Each structure also has been fabricated with 5 mm thick square frame for easier handling in transportation and during measurements. Additional material from the substrate and frames made structures more rigid and reduced the risk of warping and deformation that might have occurred during the manufacturing process and cooling of the structures.

**Figure 3:**
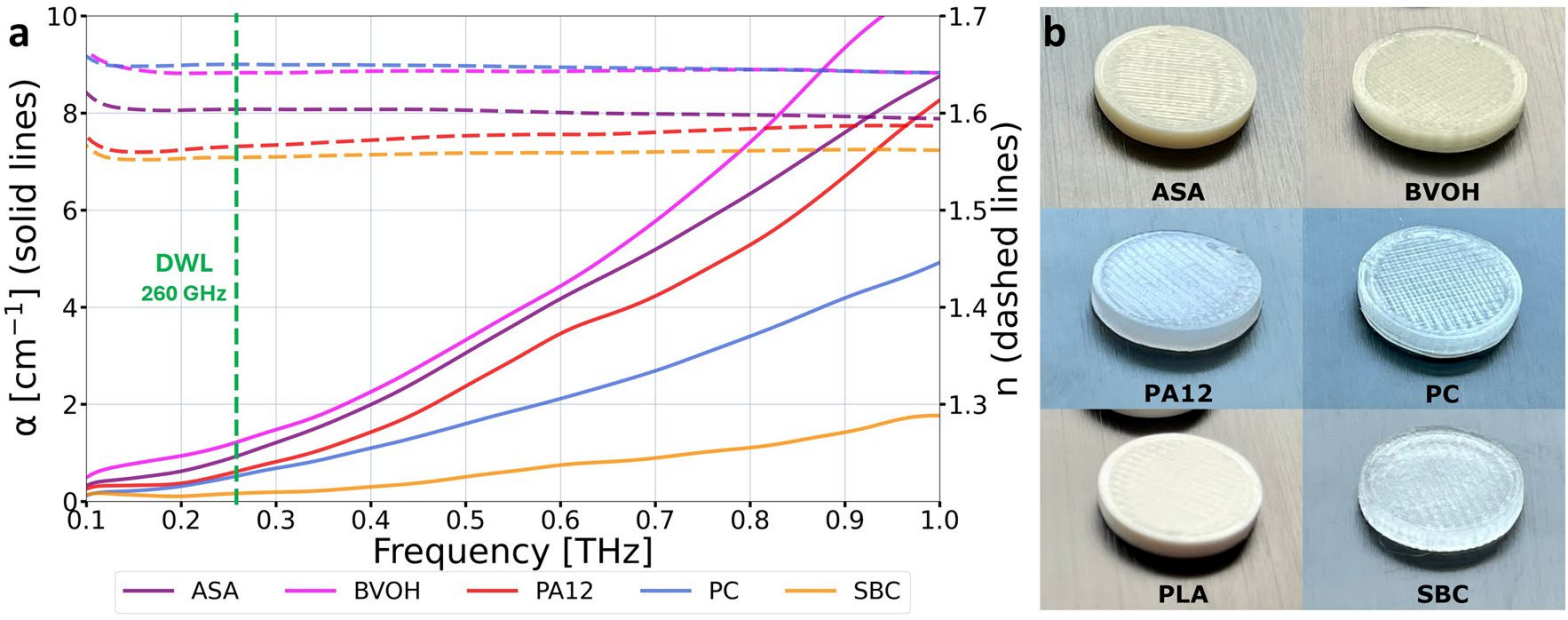
On the left (**a**): the absorption coefficients (solid lines) and the refractive indices (dashed lines) in the frequency domain for polymer materials used in the fused deposition modeling (FDM) additive manufacturing technology. The vertical dashed line marks the frequency of the source (260 GHz). The presented data was obtained by THz-TDS examination of acrylonitrile styrene acrylate copolymer (ASA), butenediol vinyl alcohol copolymer (BVOH), polyamide 12 (PA 12), polycarbonate (PC), polyactic acid (PLA), and styrene butadiene copolymer (SBC) materials. On the right (**b**): photographs of 3D printed pellets prepared for examination with THz-TDS.

Optical properties of the selection of materials had been determined with THz time-domain spectroscopy^29^ (THz-TDS TeraPusle Lx system from Teraview). After examination of multiple polymer materials available for FDM 3D printing technology^30^, presented in our study^31^, styrene butadiene copolymer (SBC) was selected for manufacturing the structures. The refractive index *n* (dashed lines) and the absorption coefficient $\alpha$ (solid lines) in the frequency domain for acrylonitrile styrene acrylate copolymer (ASA), butenediol vinyl alcohol copolymer (BVOH), polyamide 12 (PA 12), polycarbonate (PC), polyactic acid (PLA), and SBC materials are shown in Fig. 3 with photographs of the prepared samples. In Fig. 3, the vertical dashed line corresponds to the DWL frequency equal to 260 GHz for the designed structures.

The samples presented in Fig. 3b were manufactured with 450 μm horizontal resolution and 100 μm varietal resolution, which correspond to the line and layer thicknesses applied in the manufacturing process. In the THz radiation region, especially for the DWL of 1.15 mm, the lines and layers have sub-wavelength dimensions. As a result, the samples intended for THz radiation exhibit homogeneity throughout their entire volume. All the samples were manufactured the same way as the structures pretested in this study. Consequently, the measured optical characteristics of the samples are directly indicative of those of the structures. SBC, manufactured by Orbi-Tech, also called BendLay, has suitable optical properties for manufacturing phase diffractive passive optical components. According to the materials’ characteristics illustrated in Fig. 3, SBC has a significantly lower absorption coefficient than compared polymer materials in the entire verified radiation range from 100 GHz to 1 THz. The SBC refractive index value equals 1.557 and the absorption coefficient 0.162 cm^-1^ for DWL (260 GHz).

Knowing the absorption coefficient and structure thickness, one can estimate the maximum absorption (absorption at maximum thickness) by applying the Beer-Lamber’s law with the absorption coefficient as the attenuation coefficient. Such calculations yield an attenuation factor of 5.6%. Maximum losses from Fresnel reflection can be estimated from a refractive index value and under the assumption of normal incidence as 4.7% in the presented case. The total maximum losses from the material properties are then estimated at ca. 10%.

## Methods

This subsection describes a Schottky diode-based setup with frequency multipliers prepared for the experimental evaluation of manufactured structures. A short description of an experimental protocol is provided. Finally, the analyzed experimental results are summarized.

### Experimental setup and protocol

**Figure 4:**
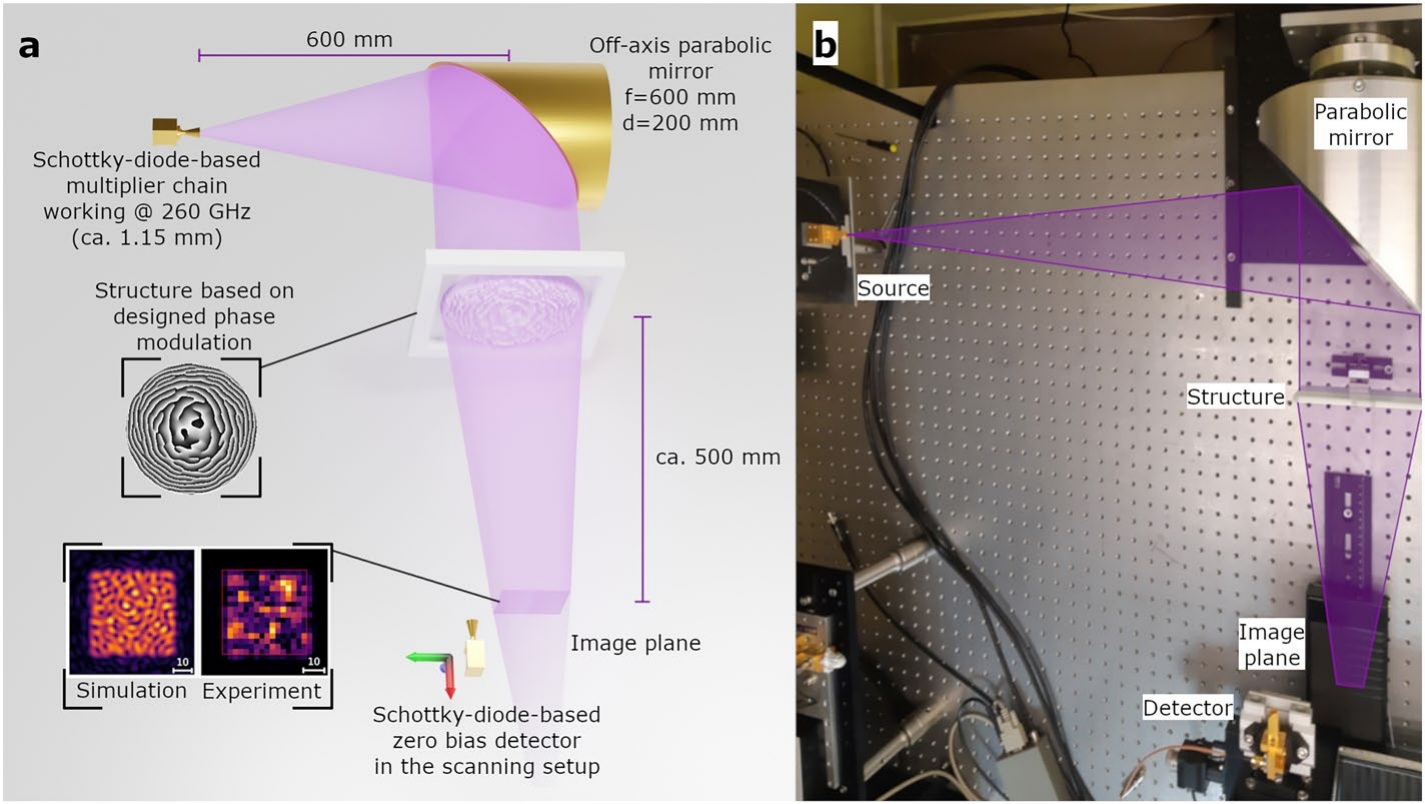
On the left (**a**): visualization of the experimental setup. The setup uses a Schottky diode-based source and detector in a single-point scanning configuration working at 260 GHz. The source illuminates the parabolic mirror from a distance equal to the mirror’s focal length. The mirror with a focal length of 600 mm and diameter of 200 mm collimates the beam and directs it at a structure under test. The beam size is approximately 200 mm in diameter and collimated beam has Gaussian-like intensity distribution. The scanning plane is placed around 500 mm behind the structure. On the right (**b**): the photograph of the setup with labeled elements and a trace of the radiation propagation.

A VDI multiplier chain based on Schottky diodes with a proper horn antenna was used as a source of radiation at the DWL (260 GHz), and a VDI WR3.4 zero bias detector (Schottky diode-based) with a symmetrical diagonal horn antenna as a detector. The DWL matched the frequency with the maximum power output of the source (0.95 mW). It has to be noted that the source is strongly coherent, which results in relatively effortless and, in many cases, unwanted interferences. Therefore, free space propagation required additional shielding/masking of the radiation reflected from different surfaces in the setup, which is still within the coherence length of the emitter. As the quasi-plane-wave illumination is necessary, an off-axis aluminum parabolic mirror was used. It redirected the collimated beam onto the investigated structure. The detector has been placed in the image plane of the evaluated DOE on three Thorlabs NRT150 motorized stages in a configuration allowing for 3D scans. Its responsivity was equal to 1500 V/W at the DWL. The voltage produced by the detector was measured with a Stanford Research SR830 lock-in amplifier. Voltage readings were directly proportional to the measured intensity for selected DWL and power ranges. The detector was moved point-by-point and line-by-line with a 2.5 mm step in a 60 mm $\times$ 60 mm square, which translated to $26 \cdot 26 = 676$ data points. The signal for a single point was averaged for 0.3 s. The scanning time of a single scan was equal to 45 min, which is connected mostly with the motion of the translation stages. It has to be noted that the selected sampling distance results in overlapping data points in the experiment due to the fact that the size of the horn is larger than the sampling distance. Figure 4 shows the visualization of the experimental setup.

A relatively large dimension of structures in relation to the propagation distance and the utilization of the scalar approach for the design procedure result in inaccuracy in the determination of the exact image plane position. In order to obtain comparable results, the best imaging distance for reference has been identified and applied for all measured structures. All structures imaged the square with significant speckles at positions roughly corresponding to the results from simulations.

### Numerical analysis

A scan of each structure provides a set of data points. Each set was normalized (rescaled) locally: the maximum from a given scan was equal to 1, and 0 remained the same (minimum values were not scaled down to 0). The normalization of the maximal registered signal was necessary for a fair comparison between the structures. It is connected with two effects. Firstly, the highest signal results from the coincidental positive interference of the radiation in the form of the brightest speckle. It is unstable and connected with the whole experimental setup; therefore, it should not influence the comparison between the investigated structures. Secondly, some of the proposed DOEs have different shapes and, thus, different active areas. This means that they gather different amounts of radiation, which further influences the values of the registered signal. The normalization of the intensity allows for independence from these factors. Region of interest (ROI), with the shape and size corresponding to the requested image, was defined as a 40 mm square. Special care was taken to keep the same number of data points in each ROI. For normalized data, two measures of central tendency (mean and median) and three measures of variability (interquartile range (IQR), sample standard deviation, and root mean square (RMS)) were calculated from ROIs. The IQR depicts the central 50% of the registered intensity values. The standard deviation denotes differences in registered intensity values in relation to the mean value in ROI. The RMS additionally accounts for the absolute intensity values of the registered data points. RMS was calculated as a square root of the ROI points’ mean square: $RMS=\sqrt{\frac{1}{n}(x_1^2+x_2^2+ \cdots +x_n^2)}$, where $x_i$ is the i-th data point from the set of ROI points, and *n* is the number of data points in the ROI set.

**Figure 5:**
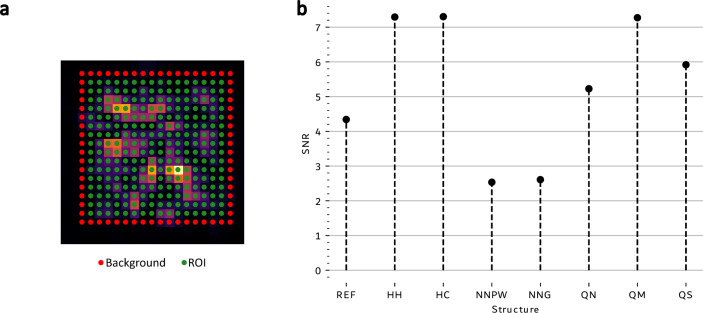
On the left (**a**): example region of interest (ROI—marked with green dots) and background (marked with red dots) are defined as a selection for calculating numerical measures. On the right (**b**): the plot of SNR for each structure.

For the calculation of the signal-to-noise ratio (SNR), an additional set of points representing the background was selected (marked with red dots in Fig. 5). Similarly as for ROI (marked with green dots in Fig. 5), background points were selected to have the same number of points for all structures. Figure 5 shows an example of ROI and background selection. SNR is calculated as the mean value of the intensity distribution from ROI data points divided by the mean value of intensity from background data points. It has to be emphasized that there are other methods to define the SNR. The prevailing approach in image processing is to use the ratio of mean signal value to standard deviation of the noise. This method is valuable for the qualification of image quality. However, this approach does not provide valuable information for beamforming tasks.

To summarize, raw data points from each experimental scan contain information on the position of the detector and measured voltage. Voltage is treated as directly proportional to the intensity of the detected radiation. From all measured data points, those corresponding to ROI and background were selected for further numerical analysis.

## Results and discussion

Figure 2 depicts all measured and simulated data together with the designed phase modulations and photographs of the manufactured structures. The last column of the figure contains a summary of properties that differ between each case. The third and fourth columns present the results of the theoretical simulations and the experimental evaluation. In both columns square areas of intensity higher than the background are observed. The difference of the intensities is significant enough to properly fit the square ROI for each structure.

From a general overview of 3D printed materials and printing methods, one can conclude that the resolution of such prints is high enough to represent most of the details from designed phase maps. The only details that may be hard to reconstruct with this printing method are wavy distortions on edges between quarters in QS and QN, which come from the propagation used in the process of constructing phase modulations in those two cases. Prints of neural-network-based structures (NNG and NNPW) have no noticeable inconsistencies due to the manufacturing method. However, some errors are expected due to significant local variations in phase modulations (large phase changes in neighboring positions).

Resolutions and sampling distances of simulations and experiments are different for each of the evaluated structures. Parameters of simulations are directly tied to parameters for the calculation of phase delay maps, whereas experimental parameters result from the experimental setup. The direct numerical comparison between simulations and experiments is difficult due to different samplings of calculation and obtained experimental matrices. A denser sampling of the calculation matrix results from the necessity of creating a high-resolution phase delay map to manufacture the structures. On the other hand, sparse sampling of the obtained experimental matrix is related to the detector’s aperture and scanning time. From the qualitative examination of simulation data, one can expect that the REF structure will be the brightest. Since NNG and NNPW were simulated with a different method (neural-network-based) than other structures, their result is hard to compare.

**Figure 6:**
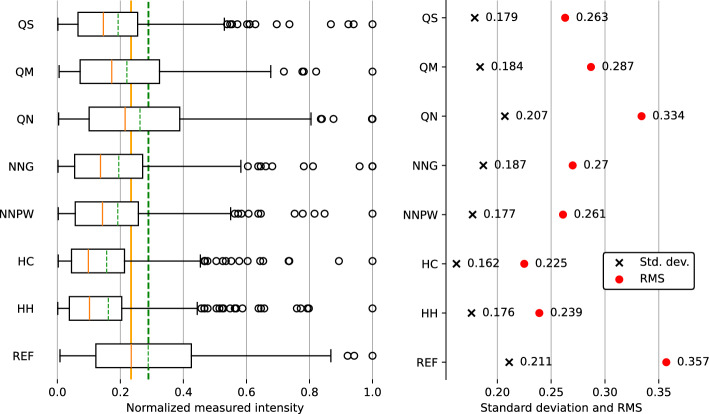
On the left: the box plot for each structure based on its region of interest (ROI) data points. Boxes represent the interquartile range (IQR) of measured intensity. Orange, solid lines inside the boxes show the median, and green, dashed lines show the mean values of registered intensity. Additionally, the highest mean and median values for reference structure have been added in the background of the box plot forming elongated lines to enable comparison with the values inside boxes for each structure. Whiskers protruding from the boxes to the left and right sides denote the lowest/highest data point within 1.5 IQR from the first/third quartile. All data points outside those ranges are displayed as empty black circles. On the right: standard deviation (denoted by black crosses) and root mean square (RMS), marked with filled red circles) from ROI. Higher values of mean and median are preferred as the distribution is less influenced by the high-intensity spots. Narrower boxes (IQR) and lower standard deviation and RMS correspond to better (lower) variability of the distribution.

Calculated numerical measures (Fig. 6) show that reference structure (REF) has the highest mean and median value (the higher, the better).

This indicates that the REF structure has the highest intensity, with QN and QM having second and third-best results in this category. Measures of variability show a slightly different picture. The least variable according to standard deviation and RMS (the lower, the better) are HC, HH, and NNPW, in that order, with HC having a significant lead. IQR (the lower, the better) indicates HC and HH as the least variable (difference at the third decimal place) with QS as the third least variable. At the same time, SNRs (displayed in Fig. 5; the higher the result, the better) show similarly high results for HH, HC, and QM.

The division into segments created different possibilities for other designs. Since each quarter has been iterated separately, QS and QM are differentiated from each other by the combination method and consequently by common (single) or separated (multiple) imaging distances. QS has better variability results than QM (3% better standard deviation, 25% IQR, and 8% RMS) but at the cost of decreased average value (by 13%) and slightly decreased SNR (by 19%).

NN-based structures (NNG and NNPW), when compared with segmented structures with the same sampling distance (QN), show improvement in variability measures (IQR by up to 31%, standard deviation by up to 14%, and RMS by up to 22%). However, NN-based structures suffer a significant reduction in average value (by at least 26%) and SNR (by at least 52%). Taking a broader look at all presented data, one can observe indications of correlation: structures manufactured with cuboid method (0.9 mm sampling distance) compared with node method structures (0.117 mm sampling distance) tend to have higher measures of central tendency, higher measures of variability, and lower SNR. This dependence is broken by NN-based structures whose measures of tendency and variability lie in between node-based structures and have the lowest calculated SNR.

Some of the presented segmentation methods use symmetric segments (HC, HH) and some asymmetric ones (e.g., QM). Symmetric approaches improve further data variability at the cost of lowering central tendency measures. At the same time, structures with symmetric segmentation keep the highest SNR value in the context of the presented structures. Different shapes of apertures do not seem to introduce significant changes.

## Conclusions

The preceding analysis shows that it is possible to obtain a greater uniformity (reduced variability) in DOEs’ output intensity distributions by an application of in-plane phase segmentation. In general, one can observe that all modified structures (non-REF) demonstrate lower variability (up to 17% in standard deviation, 45% in IQR, and 37% in RMS). However, better variability results come at the cost of the average value of the output intensity distributions, represented by measures of central tendency (up to 46% in mean values and 58% in median). Structures with the lowest variability, such as HC and HH, have at the same time the least efficient in terms of the redirected power. Among all structures, it is possible to select those that present improved variability performance with a better average/median of intensity, e.g., QS or QM. SNR plot also shows a better concentration of THz radiation on the specified area for all non-REF structures (increase up to 68%) apart from neural-network-based approaches (reduction of SNR up to 42%).

The presented holographic approach with segmentation shows improvement in the variability of intensity distribution behind designed structures. Additionally, structures still perform beamforming functions—produce square distributions at selected distances—while improving SNR. Designed structures require no active elements in the setup. On the other hand, speckle noise is still present in the output distribution, which is to be expected in setups with highly coherent sources. NN-based solutions do not show significant improvements over the presented segmentation approach. At the same time, the presented analysis does not reject the NN-based approach as futile. Further investigation in this area is required. Subsequent studies should also cover larger scanning ranges to verify the beamforming quality and, therefore, better map noise distribution outside the ROI.

The elimination of the speckle patterns in the case of highly coherent THz beams is a very challenging task. The proposed method, evaluated on the example of the uniformly illuminated square, has shown some improvements. It should be noted that the range of applications of the discussed methods covers all kinds of THz imaging or tomography systems, where speckle patterns are an issue. On the other hand, there are also applications of the speckle patterns themselves, such as, for example, ghost imaging^32,33^. Therefore, the methods of manipulating such patterns (whether to mitigate or enhance their presence) can serve as an important role in THz imaging systems.

## Data Availability

All data generated or analysed during this study are available on request from the corresponding author.
